# The 2009 pandemic H1N1 influenza vaccination in France: who accepted to receive the vaccine and why?

**DOI:** 10.1371/currents.RRN1188

**Published:** 2010-10-19

**Authors:** Jocelyn Raude, Anne-Laure Caille-Brillet, Michel Setbon

**Affiliations:** ^*^EHESP School of Public Health, Rennes - Sorbonne Paris Cité, France and ^†^EHESP, Rennes-Sorbonne Paris Cité

## Abstract

Introduction: Previous studies investigating determinants of 2009 (H1N1) pandemic influenza vaccine acceptance have focused on target groups such as healthcare workers. Few studies in the European Union have examined the self-reported reasons as well as predictive socio-demographic and health factors for pandemic influenza vaccine acceptance in the general population, even though influenza vaccine was recommended for all people.

Methods: A nationwide telephone survey was conducted in France during the peak of the outbreak that occurred in December 2009 in adults (≥ 16 years), using a proportional random-digit dialing.

Results: Interviews were completed by 1003 individuals, of whom 275 (27.4%) either had received pandemic influenza vaccine during the last weeks or intended to get vaccinated in the next weeks. Acceptance rates of pandemic vaccine were significantly higher among men, more educated and wealthier people, as well as persons who had a prior experience of influenza vaccination. The patterns of self-reported reasons for vaccine acceptance could be broadly divided into 3 groups related to (1) the mental representation of the threat – in particular the beliefs associated with the severity and personal vulnerability to the illness, (2) the perception of efficacy and safety of the vaccine, and (3) trust/distrust toward those advocating the vaccine.

Conclusions: This national study indicates that social and cognitive determinants of pandemic influenza vaccine acceptance among French adults were relatively similar to those identified by previous studies of acceptance of seasonal influenza vaccine.

## **Introduction**

In the aftermath of the emergence of the 2009 pandemic H1N1 influenza virus, large scale immunization has gradually became, in the majority of the developed countries, the primary public health measure to prevent morbidity and mortality associated with the disease.  When the first vaccines were delivered in October 2009, a high level of vaccination coverage was reached by the French government. As a precaution, enough vaccine was ordered from the pharmaceutical companies in order that 4 out of 5 persons could receive two doses. Because vaccine production would take several months to complete, a prioritization plan was announced – in which the most vulnerable people at risk (persons suffering from chronic illness, people working in health or child care-giving services, and parents of infants younger than 6 months) were invited, by letter notification, to get vaccinated first for free. In France, as in many other countries, the decision was made to provide immunizations exclusively at *ad hoc* vaccination centers for cost-effectiveness reasons. The potential benefits of pandemic H1N1 influenza vaccination for at risk groups were threefold: personal protection, protection of relatives, and reduction of absenteeism. In December, the vaccine was extended to the rest of the population. At this time, there was good evidence that the vaccine was highly effective in preventing serologically confirmed influenza, and that it had limited potential side effects [1] [2]. 

Despite considerable efforts to persuade the greatest number of people to receive the 2009 pandemic H1N1 influenza vaccine, the rate of immunization among French people was rather low: only 10% of the population had been vaccinated at the end of the campaign in January 2010 [3]. As in many countries, the relative failure of the immunization campaign against the pandemic influenza in France reminds us that the failure or success of any prevention is ultimately determined by the public acceptance of the mitigating measures deployed by the public health authorities. The aim of the present study is to better understand the reasons for accepting or refusing vaccination. The relative breakdown of the French vaccination program raises 3 critical questions: given an equal exposure to information due to intensive media coverage, were individuals of all health and social conditions equally likely to accept vaccination? If not, what kinds of individuals were most and least likely to accept vaccination? And *last but not least* why some kinds of individuals were more likely to accept vaccination? 

Toward this goal, a nationwide survey was performed during the peak of the outbreak, which occurred one month after the beginning of the immunization campaign, to identify (1) socioeconomic and health conditions that were statistically associated with vaccination against the 2009 pandemic H1N1 influenza virus and (2) to identify primary reasons that people spontaneously reported for vaccination acceptance or non-acceptance in France. Understanding the perceived barriers and benefits that affect the willingness to get vaccinated may be crucial to the development of more successful programs of immunization in the case that a novel wave of pandemic H1N1 2009 influenza should occur in the next few months.

##  **Data and methods**

The empirical data were collected in France by means of computer-assisted telephone interviews (CATI) of French adults aged 16 and over from 28 November to 18 December 2009. A proportional random digit dialing was used to select the survey participants across the country. To ensure the national representativeness of the sample, a stratified selection procedure based on the administrative area population (regions and communes) was used. Furthermore, the gender, age and occupational status of respondents were controlled by the use of quotas so that the sample approximated the last France Census data. A maximum of 5 telephone calls were performed to contact each potential respondent – mainly during evenings and weekends. Respondents were informed that the survey related to H1N1 influenza pandemic in order to obtain their verbal consent. 

Open-ended questions were asked allowing respondents to identify their reasons for accepting or refusing to be vaccinated against the 2009 pandemic H1N1 influenza. These types of questions typically provide relevant insights into common barriers to vaccination from the perspective of those being studied [4]. The survey also included several close-ended questions addressing the social and health conditions of participants: socioeconomic and demographic characteristics, experience of previous influenza vaccination, and risk factors identified by the public health authorities for the pandemic influenza (pregnancy, chronic illness, health or childcare work). Multivariable logistic regressions were performed to identify the predictive variables independently associated with the outcome variable “pandemic influenza vaccination acceptance” (self-reported intention or action of vaccination) by controlling for potential confounders. PASW for Windows Release 18.0 was used to analyze the data which were not weighted to the French population because of the use of a proportional sampling method.

## **Results**

The computer assisted telephone process resulted in 10,984 telephone contacts from which 1003 interviews were completed. Of the persons successfully contacted by the interviewers, 5,413 (50.2%) refused to participate, 4,041 (36.8%) were not eligible (due to quotas), and the remainder asked to be called back later or had comprehension problems (196 and 331, respectively). Overall, 45.9% accepted to participate in the survey. It should be noted that 41.1% of the participants reported at least one attribute that placed them in the priority group for pandemic influenza H1N1 immunization. Finally, of the 1003 participants, 7.5% [95% CI, 5.9% to 9.1%] reported to have been vaccinated and 19.9% [95% CI, 17.5% to 22.3%] the intent to get vaccinated against the 2009 pandemic H1N1 influenza virus, so that the total rate of vaccination acceptance could be estimated at more than one quarter of the population (27.4% [95% CI, 24.7% to 30.1%]).

**Table 1. Acceptance of vaccination against the 2009 pandemic H1N1 influenza virus among participants, according to socio-demographic and health-related characteristics.**

**Table:** 

**Characteristic**	**N**	**% (95% CI)**	**Total**
**Sex**			
Male	154	33.4 (29.1 to 37.7)	461
Female	121	22.3 (18.9 to 25.8)	543
**Age group**			
16-24	14	17.9 (9.4 to 26.4)	78
25-34	29	23.2 (15.8 to 30.6)	125
35-44	45	20.5 (15.2 to 25.8)	220
45-54	65	25.5 (20.2 to 30.8)	255
55-64	53	31.2 (24.3 to 38.1)	170
≥ 65	69	44.2 (36.4 to 52.0)	156
**Occupation**			
Students	10	16.7 (7.3 to 26.1)	60
Unemployed	20	20.4 (12.4 to 28.4)	98
Working	152	24.4 (21.1 to 27.7)	623
Retired	93	42.1 (35.6 to 48.6)	221
**Education**			
Low	92	25.4 (21.0 to 29.8)	362
Intermediate	46	23.1 (17.3 to 28.9)	199
High	137	31.0 (26.7 to 35.3)	442
**Monthly Income**			
< €3,000	134	23.7 (20.2 to 27.2)	565
€3,000 - €4,500	59	28.6 (22.5 to 34.7)	206
> €4,500	82	32.2 (26.5 to 37.9)	255
**Risk factors**			
Yes	62	36.8 (29.5 to 44.1)	168
No	213	25.7 (22.8 to 28.6)	829
**Parental status**			
Yes	13	33.3 (18.5 to 48.1)	39
No	262	27.2 (24.5 to 29.9)	963
**Working in health or child care**			
Yes	81	30.8 (25.3 to 36.3)	263
No	194	26.2 (23.1 to 29.3)	740
**Previous influenza vaccine receipt**			
Yes	156	41.7 (36.8 to 46.7)	374
No	119	18.9 (15.9 to 21.9)	630
**Total**	**275**	**27.4 (** **24.7 to 30.1)**	1004

** **
***Predictive factors of vaccine acceptance*** As indicated in table 1, rates of vaccination acceptance did not differ substantially according to the familial (whether respondent had children younger than 6 years of age) and professional status (whether respondents worked in health or childcare services), or the level of education of the participants. By contrast, the health status, income, gender, age, and previous influenza vaccine receipt of the respondents were significantly associated with the reported vaccination acceptance (*p* > 0.05). However, after adjusting for other socioeconomic or health variables, we only found that vaccination acceptance was higher among men than women (adjusted OR = 1.61, *p* = 0.003), higher among more educated people than less educated people (adjusted OR = 1.20, *p* = 0.047), and higher among wealthier people than poorer people (adjusted OR = 1.24, *p* = 0.024). The participants who had an experience with influenza vaccination in previous years (adjusted OR = 2.36, *p* < 0.001), as well as those who were working in health or child care (adjusted OR = 1.54, *p* = 0.013), were also significantly more likely to accept vaccination against the 2009 pandemic H1N1 influenza. **Table 2. Categories of reasons reported by participants for accepting vaccination against the 2009 pandemic H1N1 influenza.**

**Table:** 

**Categories of self-reported reasons**	**N**	**%**
Self-protection – including perception of personal risk factors	126	45.8
Protection of significant others (parents, children, friends, etc.)	77	28.0
Protection of patients or colleagues – including work ethic	35	12.7
Trust in the vaccine; compliance with recommendation	25	9.1
Fear of change in the nature of the disease	6	2.2
Other reasons	6	2.2
**Total**	**275**	**100.0**

** **
**Table 3. Categories of reasons reported by participants for refusing vaccination against the 2009 pandemic H1N1 influenza.**

**Table:** 

**Categories of self-reported reasons**	** **	**N**	**%**
Belief that the vaccine is dangerous, fear of adverse reactions		218	35.2
Belief that pandemic influenza is a minor illness, lack of perception of own risk		173	27.9
Belief that the vaccine is not effective		73	11.8
Belief that he or she has already contracted the disease		54	8.7
Preference for alternative methods of protection		40	6.5
Distrust of media, pharmaceutical companies or public authorities (including belief in conspiracy theories)		38	6.1
Medical or lay recommendation against vaccination		15	2.4
Other reasons		8	1.3
**Total**	** **	**619**	**100.0**

** **  ***Reported reasons for vaccine acceptance*** The primary motives for acceptance or non-acceptance of the 2009 pandemic H1N1 influenza vaccine reported by the respondents in the survey are presented in tables 2 and 3 respectively. As indicated in table 2, the most frequent reasons that participants reported for accepting vaccination against the pandemic influenza included mainly protection of self or significant others (members of family, friends, patients or colleagues) and trust in the vaccine or biomedical community. Unsurprisingly, participants that were identified as persons at higher risk by the public health authorities were substantially more likely than the other participants to cite self-protection as a primary motive for vaccination acceptance (62.9% *vs.* 40.8%, *x*² = 9.4, *p* = 0.002). On the other hand, the major reasons reported by the respondents for reluctance to vaccinate (table 3) included perceived risk of potential adverse effects, belief that the pandemic influenza was a benign illness, and belief that the vaccine was ineffective or useless. Interestingly, respondents that belonged to higher risk groups for the pandemic H1N1 influenza were more likely than other reluctant respondents to report that they feared side effects from the vaccine (38.8% *vs.* 28.6% respectively, *x*² = 15.8, *p* < 0.05).   

## **Discussion**

In this study, we investigated the level and distribution of vaccination acceptance against the 2009 H1N1 influenza virus during the peak of the epidemic in France. This provided an exceptional opportunity to investigate the impact of social and health conditions on vaccination behavior in the context of spreading of an emerging infectious disease. To the best of our knowledge, few studies have examined the role of these factors in the epidemiology of pandemic A (H1N1) influenza immunization in the general population. On the basis of this telephone survey performed in December 2009, the rate of vaccination acceptance, which includes self-reported intentions or actions of immunization, could be estimated at about one quarter (27.4%) of the French population ≥ 16 years of age. More precisely, actual vaccination against the pandemic H1N1 influenza virus was estimated at 7.5%, which is congruent with the more or less 10% estimate of the vaccination rate calculated by the French Ministry of Health from the number of persons who received the vaccine in the vaccination centers for the immunization period 2009-10 [2]. Concerning the socio-demographic distribution of vaccination acceptance among French adults, our data have shown that the male, older and more advantaged participants – who are characterized by higher educational and material resources – were more likely to accept the vaccine. Surprisingly, with the notable exception of sex, these results are quite comparable to those of many epidemiological surveys conducted in developed countries about seasonal influenza vaccination [5] [6] [7] [8] [9]. Indeed, a large range of socioeconomic variables such as age, education level and income have consistently been demonstrated to influence influenza vaccination behaviors. Nevertheless, after controlling for other potential confounding variables, we found that vaccination acceptance was much more determined by individuals’ experiences of seasonal influenza vaccination in previous years than by age, which is consistent with the available data from various surveys related to pandemic influenza [9] [10].

Noticeably, vaccination acceptance was not considerably higher among the participants belonging to the categories that were identified as priority groups for pandemic vaccination by the public health authorities. As indicated in table 1, only the respondents affected by chronic diseases or pregnancy were found to report a significantly higher rate of vaccination acceptance. To further examine the influence on these factors on vaccination acceptance, a simultaneous logistic regression was performed to control for other potentially confounding socio-demographic variables. Interestingly, health condition dropped out as a predictor, suggesting that risk factors are perhaps only indirectly associated with attitude toward vaccination through the influence of a range of socio-demographic variables such as age or occupational status. On the contrary, professional status was found to significantly affect vaccination acceptance after adjusting for other socio-demographic variables. This might be attributable to the fact that those working in the health or childcare fields were subjected to more immediate and personal pressures from their social and institutional environment. Nevertheless, the effects of social conformity remained relatively weak since less than one third of members of this key target group were likely to accept vaccination. Overall, the rate of acceptance of pandemic influenza vaccination among health and childcare workers was not substantially different from those observed for seasonal influenza in previous years in European countries [11] [12] [13].

At this stage, it remains necessary to uncover the reasons why certain persons or groups were more likely to accept immunization against the 2009 pandemic H1N1 influenza virus than others, even when socio-demographic variables, as well as risk factors, were controlled for. In the recent literature, one of the dominant strategies has been to use open-ended questions within large-scale surveys. This type of questioning is known to encourage more complete and meaningful answers in surveys by permitting subjects to express their motivations and/or sentiments in their own phrasing [4]. In the present study, the primary reasons associated with acceptance and rejection of vaccination can be broadly divided into 3 groups. The first group addresses the mental representation of the threat – in particular the beliefs and judgments related to the severity of the illness and risk factors (i.e., which groups are more vulnerable to the infection), as well as the emotional response to the risk of contracting the disease. The second relates to the perception of the vaccine, including concerns about its safety or effectiveness. The third group of motives can be associated with issues of trust toward those advocating the vaccine (the government, the pharmaceutical industry, or biomedical experts) – including beliefs in conspiracy theories. 

Eventually, the motivations that underlie the acceptability of vaccination could be approached to a large extent from a traditional risk/benefit analysis. According to this basic model of decision-making under uncertainty, the acceptance or rejection of any protective action should be interpreted as the outcome of a trade-off between the risks and personal or societal benefits associated with products or activities that are recognized for their preventive value. From this theoretical perspective, the public acceptability of vaccination is expected to vary roughly as a direct function of both perceived risk and perceived benefit. Moreover, the perceived benefit associated with immunization depends in turn, for a large part, on the perceived risk of contracting the disease – which is generally known to result from two main cognitive components: the personal likelihood of becoming infected (vulnerability) and the seriousness of the consequences of that infection (severity). Although the relevance of the risk/benefit approach would deserve a more complex discussion that is beyond the scope of this article, most of the primary reasons that were spontaneously invoked by the respondents might indicate that people actually perform a sort of trade-off between the perceived risk and the perceived benefit associated with the vaccine uptake. 

For example, among the principal reasons for the non-acceptance of vaccination were (1) belief that the vaccine against the 2009 pandemic H1N1 influenza virus is dangerous or ineffective and (2) belief that the pandemic influenza is not a serious illness, suggesting that the perceived risk exceeds the perceived benefit associated with pandemic influenza vaccination. Thus, almost half of the reluctant respondents (47.0%) indicated that they had doubts about either safety or efficacy of the pandemic vaccine, and more than one quarter (27.9%) felt that the pandemic H1N1 influenza could be considered to be a minor illness. Finally the motives captured by open-ended questions provided results that are largely consistent with those of previous quantitative and qualitative studies on the determinants of seasonal influenza vaccine acceptance. According to certain reviews of literature, the fear of side effects can be easily identified as one of the most commonly reported barriers to vaccination across all communicable diseases [14] [15] [16].

Several limitations can be pointed out in this study. First, even if the sampled population did not significantly differ from the general population in terms of age, gender, occupation and place of residence, it cannot be ruled out that the subjects who refused to participate in our study were characterized by a range of psychological and/or sociological attributes that made them different from the cooperating subjects with regards to vaccination acceptance or non-acceptance. Second, the timing of the survey (early December) might have led to both an underestimate of the vaccination coverage rate, and an overestimate of vaccination intention among the French adult population since the controversy about the safety and/or necessity of the pandemic H1N1 influenza vaccination was growing over time. As noted above, the actual vaccination coverage rate was assessed at the end of the domestic outbreak by the Ministry of health and found to include approximately 10% of the French. Third, the nature of reasons offered by the participants to justify their acceptance or refusal to get vaccinated against the pandemic influenza might have been subject to possible biases of social desirability (or conformity). This typical bias – which describes the subjects’ propensity to report a range of reasons for a particular behaviour that are different from their “real” motivation because they are viewed as more socially acceptable – has been extensively well-documented in the methodological literature [4] [17].

To conclude, the results of this study tend to show that the 2009 pandemic H1N1 influenza was not perceived by the French population to be significantly different from the seasonal influenza. Notably, the distribution and justification of vaccination acceptance among the participants were strongly similar to those of seasonal influenza vaccination acceptance [18] [19], and consequently contributed to the maintenance of health inequalities in France. In this regard, it should be noted that the pandemic H1N1 influenza has been commonly and ironically named “grippette” (little flu) in France. Thus, as long as the patterns of mortality and/or morbidity associated with the pandemic influenza did not differ crucially from those related to the recent seasonal influenza, it would probably have been difficult to observe substantially higher vaccination coverage rates for this emerging infectious disease among the various key subgroups within the French population. In terms of risk communication, these results have one major implication: the failure of the immunization campaign cannot be solely attributed to the controversy about the potential side effects of the vaccine, but also to the nature and level of the perceived risk associated with the pandemic H1N1 influenza. From this point of view, a public health information campaign that primarily focuses on convincing people of the safety of the vaccine would likely be less fruitful than expected.

## **Funding information**

 This research was funded by a grant from the Service d’Information du Gouvernement (French Government Office for Information) which was in charge of the public communication related to the  2009 H1N1 pandemic influenza in France.  

## **Competing interests**

The authors have declared that no competing interests exist

## References

[ref-1197955208] Center for Disease Control. Preliminary results surveillance for Guillain-Barré syndrome after receipt of influenza A (H1N1) 2009 monovalent vaccine - United States, 2009-2010. Morbidity and Mortality Weekly Report. 2010; 59(21):657-61. 20520590

[ref-4109011404] République Française. Grippe A(H1N1) : l'épidémie recule mais un second pic pandémique reste possible. January 15, 2010. (Accessed June 10, 2010, at http://www.gouvernement.fr/gouvernement/grippe-ah1n1-l-epidemie-recule-mais-un-second-pic-pandemique-reste-possible).

[ref-431708491] PoteVellozzi C, Burwen DR, Dobardzic A, Ball R, Walton K, Haber P. Safety of trivalent inactivated influenza vaccines in adults: background for pandemic influenza vaccine safety monitoring. Vaccine. 2009;27:2114-20. 10.1016/j.vaccine.2009.01.125 19356614

[ref-2599719425] Aday, L.A. Designing And Conducting Health Surveys: A Comprehensive Guide. San Fransisco: Jossey-Bass, 2006.

[ref-3208703582] Rehmet S, Ammon A, Pfaff G, Bocter N, Petersen LR. Cross-sectional study on influenza vaccination, Germany, 1999-2000. Emerg Infect Dis. 2002;8(12):1442-47. 10.3201/eid0812.010497 PMC2738508 12498661

[ref-497165168] Chapman GB, Coups EJ. Predictors of influenza vaccine acceptance among healthy adults. Prev Med (1999) 29:249–62. 10.1006/pmed.1999.0535 10547050

[ref-776823639] Jones TF, Ingram LA, Craig AS, Schaffner W. Determinants of influenza vaccination, 2003-2004: shortages, fallacies and disparities. Clin Infect Dis. 2004;39(12):1824-28. 10.1086/427153 15578406

[ref-1631353622] Endrich MM, Blank PR, Szucs TD. Influenza vaccination uptake and socioeconomic determinants in 11 European countries. Vaccine 2009;27(30):4018-24. 10.1016/j.vaccine.2009.04.029 19389442

[ref-1432618043] Setbon, M, Raude, J. Factors in vaccination intention against the pandemic influenza A/H1N1. European Journal of Public Health (Access published online on May 5, 2010). 10.1093/eurpub/ckq054 PMC7314046 20444821

[ref-12391222] Maurer J, Harris KM, Parker A, Lurie N. Does receipt of seasonal influenza vaccine predict intention to receive novel H1N1 vaccine: evidence from a nationally representative survey of U.S. adults. Vaccine (2009) 27:5732–4. 10.1016/j.vaccine.2009.07.080 PMC2771376 19679219

[ref-1933867124] Maltezou HC, Maragos A, Katerelos P, Paisi A, Karageorgou K,Papadimitriou T, et al. Influenza vaccination acceptance among health-care workers: a nationwide survey. Vaccine. 2008;26(11):1408-10. 10.1016/j.vaccine.2008.01.049 18313179

[ref-550675356] Leitmeyer K, Buchholz U, Kramer M. Influenza vaccination in German health care workers: effects and findings after two rounds of a nationwide awareness campaign. Vaccine 2006;24(47–48):7003–8. 10.1016/j.vaccine.2006.04.040 16730866

[ref-931629878] Rothan-Tondeur M, deWazieres B, Lejeune B, Gavazzi G. Influenza vaccine coverage for healthcare workers in geriatric settings in France. Aging Clin Exp Res, 2006;18(6):512–6. 10.1007/BF03324852 17255641

[ref-3983947584] Hollmeyer HG, Hayden F, Poland G, Buchholz U. Influenza vaccination of health care workers in hospitals: a review of studies on attitudes and predictors. Vaccine 2009;27(30): 3935-44. 10.1016/j.vaccine.2009.03.056 19467744

[ref-287228118] Mills E, Jadad AR, Ross C, Wilson K. Systematic review of qualitative studies exploring parental beliefs and attitudes toward childhood vaccination identifies common barriers to vaccination. J Clin Epidemiol. 2005;58(11):1081-88. 10.1016/j.jclinepi.2005.09.002 16223649

[ref-496327617] Streefland P, Chowdhuryb AMR, Ramos-Jimenezc P. Patterns of vaccination acceptance. Social Science & Medicine. 1999;49(12):1705-1716. 10.1016/s0277-9536(99)00239-7 10574240

[ref-3865927065] Tourangeau R, Yan T. Sensitive questions in surveys. Psychological Bulletin. 2007; 133(5): 859-883. 10.1037/0033-2909.133.5.859 17723033

[ref-2636294724] Müller D, Szucs TD. Influenza vaccination coverage rates in 5 European countries: a population-based cross-sectional analysis of the seasons 02/03, 03/04 and 04/05. Infection. 2007;35(5):308-19. 10.1007/s15010-007-6218-5 17885730

[ref-620217496] Groupe d’Expertise et d’Information sur la Grippe (GEIG). Le taux de couverture vaccinale en France. (Accessed June 10, 2010, at http://grippe-geig.com/fr/vaccination/couverture_vaccin.php.).

